# Spherical Lactic Acid Bacteria Activate Plasmacytoid Dendritic Cells Immunomodulatory Function via TLR9-Dependent Crosstalk with Myeloid Dendritic Cells

**DOI:** 10.1371/journal.pone.0032588

**Published:** 2012-04-10

**Authors:** Kenta Jounai, Kumiko Ikado, Tetsu Sugimura, Yasuhisa Ano, Jonathan Braun, Daisuke Fujiwara

**Affiliations:** 1 Central Laboratories for Frontier Technology, Kirin Holdings Co., Ltd., Kanazawa, Yokohama, Japan; 2 Technical Development Center, Koiwai Dairy Foods Co., Ltd., Sayama, Japan; 3 Department of Pathology and Laboratory Medicine, David Geffen School of Medicine, University of California Los Angeles, Los Angeles, California, United States of America; National Jewish Health and University of Colorado School of Medicine, United States of America

## Abstract

Plasmacytoid dendritic cells (pDC) are a specialized sensor of viral and bacterial nucleic acids and a major producer of IFN-α that promotes host defense by priming both innate and acquired immune responses. Although synthetic Toll-like receptor (TLR) ligands, pathogenic bacteria and viruses activate pDC, there is limited investigation of non-pathogenic microbiota that are in wide industrial dietary use, such as lactic acid bacteria (LAB). In this study, we screened for LAB strains, which induce pDC activation and IFN-α production using murine bone marrow (BM)-derived Flt-3L induced dendritic cell culture. Microbial strains with such activity on pDC were absent in a diversity of bacillary strains, but were observed in certain spherical species (*Lactococcus*, *Leuconostoc, Streptococcus and Pediococcus*), which was correlated with their capacity for uptake by pDC. Detailed study of *Lactococcus lactis* subsp. *lactis* JCM5805 and JCM20101 revealed that the major type I and type III interferons were induced (IFN-α, -β, and λ). IFN-α induction was TLR9 and MyD88-dependent; a slight impairment was also observed in TLR4^-/-^ cells. While these responses occurred with purified pDC, IFN-α production was synergistic upon co-culture with myeloid dendritic cells (mDC), an interaction that required direct mDC-pDC contact. *L. lactis* strains also stimulated expression of immunoregulatory receptors on pDC (ICOS-L and PD-L1), and accordingly augmented pDC induction of CD4^+^CD25^+^FoxP3^+^ Treg compared to the *Lactobacillus* strain. Oral administration of *L. lactis* JCM5805 induced significant activation of pDC resident in the intestinal draining mesenteric lymph nodes, but not in a remote lymphoid site (spleen). Taken together, certain non-pathogenic spherical LAB in wide dietary use has potent and diverse immunomodulatory effects on pDC potentially relevant to anti-viral immunity and chronic inflammatory disease.

## Introduction

Dendritic cells (DC) are a crucial immune cell subset linking innate immune response and acquired immunity, by their distinct capacity to recognize pathogenic and endogenous inflammatory signals. DC are comprised of numerous populations that vary in their tissue distribution, pattern of cytokines/chemokine production, and their interactions with other immune cells. However, at the simplest level they DC are subdivided into plasmacytoid dendritic cell (pDC), myeloid dendritic cell (mDC) and CD8^+^ dendritic cell (CD8^+^DC). pDC is a rare subset representing less than 1% of murine spleen and lymph node cells [1] and human peripheral blood mononuclear cells [2]. They are notable for their distinct use of certain toll-like receptor (TLR) families that sense the presence of bacteria and viruses. In particular, TLR9, which recognizes microbial nucleic acids by detecting unmethylated CpG motifs of DNA, and TLR7, that sense microbial RNA or synthetic guanosine analogs [3], are endosomal receptors that are highly expressed in pDC, and lead to their production of type I interferons (IFN).

The type I IFN family includes IFN-α and IFN-β which serve as a first-line defense in infection and prime both innate and adaptive immune responses [4]–[6]. Other IFNs, such as IFN-β, IFN-γ and IFN-λ are also important for anti-viral immunity [7], [8] notably as products of macrophages and TNF or iNOS producing DC [9], monocyte-derived DC [10] and pDC in response to viruses and TLRLs in humans [11] and mice [12]. Type I IFN blocks viral replication by inducing a series of proteins such as MxA, an IFN*α*-inducible intracellular protein [13], oligoadenylate synthetase, and double-stranded RNA-(dsRNA-)-dependent protein kinase (PKR). Accordingly, they have formed the basis for biologic therapy in a number of viral infectious diseases [14]. Furthermore, type I IFN also trigger differentiation and maturation of mDC [15], modulation of CD8^+^T cells [16], activation of Th1 [17], NK cells [18], and induction of primary response of antibodies [19], [20]. Thus, agonistic agents of pDC or type I IFN itself are anticipated to be therapeutically useful in cancer and allergy therapy.

While type I IFN induction is most associated with viral infection, several taxa of pathogenic bacteria, such as *Chlamydia* [21], *Salmonella* [22], *Mycobacteria* [23], [24], *Listeria* [25], and *Staphylococcus aureus* [26] elicit IFN-α production in humans and mice, the latter mediated by pDC [27]. Nonpathogenic microbiota have been less studied regarding DC-mediated IFN induction. A category of such microbiota of interest are lactic acid bacteria (LAB), used in commercial probiotic preparations, and consumed in variety of foods, such as yogurt, dietary supplement, pickles, alcohol beverages, and soft drinks. One of their well-documented impacts on immunity of LAB is an ability to skew an allergic Th2 response toward a Th1 phenotype via eliciting IL-12 production from macrophages and mDC [28]–[32]. Previously, we have reported that IL-12 inductive activity of LAB is highly diverse depending on species and strain [32]. LAB strains with the most potent inducing activity for IL-12 appear to prevent allergic symptoms, based both on mouse models [33] and a human clinical trial [34]. An additional intriguing function of LAB is induction of the anti-inflammatory cytokine, IL-10. Allergic symptoms are ameliorated or prevented by consumption of specific LAB strain in humans via induction of IL-10 [35]–[37].

The cell types involved in these immunologic responses to LAB are incompletely understood, and studies thus far have focused on macrophages and mDC. pDC, notable for their production of type I IFNs, may have an unappreciated role in these LAB-host interactions. In the present study, we identify a subset of non-pathogenic LAB highly inducing for IFN-α, IFN-β and IFN-λ. We also provide evidence for their pDC-dependent induction of Treg, and a surprising cross-talk of mDC in these LAB-induced pDC roles. Furthermore, oral intake of one of the selected strains of *L. lactis* JCM5805 significantly increased pDC activation markers in mesenteric lymph node (MLN), a direct draining site for dietary LAB and their products. These data provide new insights into the immunologic profile of the host response to LAB strains, which may be pertinent to their utility as a host modifier in viral infection and cancer.

## Materials and Methods

### Mice

Eight to ten weeks old female C57BL/6J wild-type, TLR2^-/-^, TLR4^-/-^, TLR7^-/-^, TLR9^-/-^ and MyD88^-/-^ were purchased from Charles River Japan.

### BM-derived DC Culture

Flt-3L induced DC were generated as previously described [38]. In short, BM cells were extracted from C57BL/6J, and erythrocytes were removed by brief exposure to 0.168 M NH_4_Cl. Cells were cultured at a density of 5×10^5^ cells/ml for 7 days in RPMI1640 medium supplemented with 1 mM sodium pyruvate (Invitrogen), 2.5 mM HEPES (Invitrogen), penicillin-streptomycin (Invitrogen), 50 µM 2-ME (Invitrogen), 10% FCS and 100 ng/ml Flt-3L (R&D systems). LAB were added at the concentrations of 10 µg/ml, and cultures were continued for 48 hrs. 1 µg/ml of Pam_3_CSK_4_ (invivogen), 10 µg/ml of Poly(I:C) (invivogen), 5 ng/ml of LPS (Sigma), 0.1 µM of CpG-A (invivogen) and 10 µg/ml of lipoteicoic acid (invivogen) were used as positive controls.

### LAB Strains

LAB strains tested in this study were purchased from the collections held at Japan Collection of Microorganisms (JCM), Institute for Fermentation Osaka (IFO), Tokyo University of Agriculture Culture Collection Center (NRIC), American Type Culture Collection (ATCC), NITE Biological Resource Center (NBRC) and DANISCO. Cultures of LAB strains were grown at 30^o^C or 37^o^C for 48 hrs in MRS broth (OXOID) or GAM broth (Nissui) or M17 broth (OXOID) according to instructions. Cultured LAB strains were washed twice with sterile distilled water, heat-killed at 100^o^C, lyophilized and suspended in PBS. Live organisms were also tested. Live or heat-killed LAB cell numbers were calculated by using a particle counter (CDA-1000X, Sysmex).

### Antibodies

The following fluorescence-conjugated anti-mouse mAbs were purchased from eBioscience: B220 (RA3-6B2), CD11c (N418), MHC Class ΙΙ (M5/114.15.2), CD40 (HM40-3), CD80-APC (16–10A1), CD86 (GL1), PD-L1 (M1H5), ICOS-L (HK5.3), CD25 (PC61.5), FoxP3 (FJK-16s), CD4 (L3T4) and IFN-γ (XMG1.2). CD3ε (145-2C11) and CD11b (M1/70) were from BD Pharmingen. Anti-mPDCA-1 (JF05-1C2.4.1) was purchased from Miltenyi Biotec.

### FACS Analysis

Cells for FACS analysis were stained with fluorescent dye-conjugated Abs (FITC, PE, PerCP, APC, PE-Cy7, APC-Cy7). After staining, the cells were washed twice with FACS buffer (0.5% BSA in PBS buffer) and suspended in 2% paraformaldehyde for FACS analysis. Data were collected by FACS Cant II (BD Biosciences) and analyzed by FCS Express software (De Novo Software) for % positive cells and expression levels of gated cells. BM-derived Flt-3L induced pDC and mDC was defined as CD11c^+^CD11b^-^B220^+^ and CD11c^+^CD11b^+^B220^-^, respectively.

### Purification of pDC and mDC

pDC and mDC from BM-derived Flt-3L induced DC culture were sorted by FACS Aria (BD Biosciences). Purity of sorted pDC or mDC was more than 95%.

### Isolation and Purification of DNA from LAB Strains

Total DNA was prepared as described previously [39].

### ELISA

Mouse Interferon Alpha ELISA Kit and Mouse Interferon beta ELISA Kit were purchased from PBL Biomedical Laboratories. Mouse IFN-γ Ready-SET-GO, Mouse TNF-α Ready-SET-GO and Mouse IL-28/IFN-λ Ready-SET-GO kit were purchased from eBiosciences. Mouse IL-12 (p40) ELISA set was purchased from BD Pharmingen.

### Transwell Culture Assay

Purified pDC and mDC were cultured using Transwell filter (0.4 µm in pore diameter; Costar) at a density of 1×10^5^ cells/ml in various conditions as below. (1) Purified pDC were seeded in conditioned medium (denoted pDC). (2) Purified mDC were seeded in conditioned medium (indicated as mDC). (3) 1×10^5^ cells/ml of purified pDC and mDC were mixed at the ratio of 1∶1 in conditioned medium (denoted pDC+mDC). (4) Purified pDC and mDC were seeded in the upper chamber (inserts) and the bottom chamber, respectively (denoted pDC/mDC). (5) Purified mDC and pDC were seeded in the upper chamber and the bottom chamber, respectively (denoted mDC/pDC), and CpG-A or LAB (0.1 µM and 10 µg/ml, respectively) were added to the upper chamber or medium and cultured for 48 hrs at 37^o^C. Culture supernatants were assayed for concentrations of IFN-α, IL-12 and TNF-α.

### Microscopic Observation of Activation of DC and Incorporation of LAB Into DC

To observe morphology of pDC stimulated by LAB, sorted pDC were stimulated with LAB for 48 hrs, attached to glass slides (Matsunami glass Ind., Ltd) using Cytospin (Thermo Scientific), and stained by Diff-Quick (Sysmex) according to the manufacturer’s protocol.

To assess uptake of LAB, fluorescent LAB were produced by suspending LAB at 1 mg/ml in a FITC solution (FITC isomer 1 (Sigma) dissolved in 0.1M NaHCO_3_ pH9.0 buffer at 0.1 mg/ml), and incubated for 60 min at 25^o^C. LAB were washed three times with PBS and yielded FITC-labeled LAB. BM-derived purified pDC were placed onto glass coverslips (Matsunami Glass Industries, Ltd.). FITC-labeled LAB (10 µg/ml) were added to coverslips bearing pDC and incubated for 24 hrs at 37^o^C. Subsequently, the coverslips were washed with PBS and stained with anti-B220- PE-Cy5.5 (e Bioscience) for 1 hr. Fluorescent microscopic observation was performed using a OLYMPUS BX60.

### Mixed Lymphocyte Reaction

Splenic naïve CD4^+^T cell were prepared from BALB/c with CD4^+^CD62L^+^ T cell isolation kit II (Miltenyi Biotec) according to manufacturer’s instructions. DCs were induced from C57BL/6 BM cells and pDC were sorted with a FACS Aria. Purified pDC were stimulated with 10 µg/ml of *L. lactis* JCM5805 or *L. rhamnosus* ATCC53103 or 0.1 µM of CpG-A overnight. Then, pDC were treated with 10 µg/ml of mitomycin C (Dojindo) for 30 min at 37^o^C and co-cultured with 5×10^5^ cells/ml of BALB/c naïve CD4^+^T cell in RPMI medium supplemented with or without 0.75 ng/ml of human TGF-β (Peprotech) for 7 days. Cells cultured in the presence of TGF-β were collected and washed with PBS, then stained for CD3, CD4 and CD25. Next, cells were treated using FoxP3 staining buffer set (eBioscience) according to manufacturer’s instructions and were stained for intracellular FoxP3. Cells were gated on CD3^+^CD4^+^, and then evaluated for CD25 and FoxP3 expression. Cells cultured in the absence of TGF-β were stained for CD3, CD4, then fixed by Cytofix/Cytoperm kit (BD biosciences) according to manufacturer’s instructions, and finally stained for intracellular IFN-γ.

### *In Vivo* Response to L. Lactis JCM5805

6 weeks-old female C57BL/6 mice were acclimatized for 1 week with free access to water and a basic diet, AIN93G (Oriental Yeast, Tokyo, Japan). Mice were divided into two groups, each group consisting of eight mice. Control groups were fed AIN93G, and test groups were fed AIN93G to achieve 1 mg/ mouse/day of *L. lactis* JCM5805. Two weeks later, mice were sacrificed, and spleen and MLN were excised. To analyze DCs status, low density cells fractions were prepared as described previously with some modifications [38]. Spleen and MLN were minced in Mg^2+^- and Ca^2+^-free HBSS and digested with 1 mg/ml collagenase (Sigma) and 0.2 mg/ml DNase I for 30 min at 37^o^C. EDTA was adjusted to 30 mM and incubated for 10 min at room temperature. Tissue lysates were filtered through a 100 µm nylon cell strainer and layered onto 15% Histodenz (Sigma) in RPMI1640 containing 10% FCS, and centrifuged at 450×g for 20 min without braking. Low-density cells at the interface were collected, stained for CD11c, CD11b, mPDCA-1, CD86 and MHC class II), and analyzed for CD86 and MHC class II expression on pDC and mDC by FACS Canto II. pDC and mDC were defined as CD11c^+^CD11b^-^mPDCA-1^+^ and CD11c^+^CD11b^+^mPDCA-1^-^, respectively. Animal procedures and experiments were approved by Laboratory Animal Care Committee of Central Laboratories for Frontier Technology, Kirin Holdings Co., Ltd. The approval ID of experiments is YO1000035.

### Statistics

Results were evaluated by standard student’s t test.

## Results

### Screening of LAB Strains which Induce IFN-α Production from BM-derived DC

We first screened for LAB species and strains that with the capacity to stimulate DC. As listed in Table S1 (rod-shaped strains) and Table S2 (spherical strains), 125 LAB strains representing 31 species were tested using BM-derived Flt-3L induced DC cultures. BM-derived Flt-3L culture yields a mixture of pDC (CD11c^+^CD11b^-^B220^+^) and mDC (CD11c^+^CD11b^+^B220^-^). 10 µg/ml of heat-killed LAB strains were added to Flt-3L induced DC culture and IFN-α concentrations were determined from culture supernatants. Significant IFN-α production (>50 pg/ml) was induced by only a minority of LAB strains: none of 90 rod-shaped LAB, and only 13 strains of 35 spherical strains (Table 1). These 13 strains included members of the *Lactococus*, *Leuconostoc*, *Streptococcus*, and *Pediococcus* genera. The most potent stimulators, *Lactococcus lactis subsp. lactis* JCM5805 and JCM20101, were selected for more detailed study.

**Table 1:** List of strains with significant production of IFN-α.

Strain ID	Genera	Production of IFN-α (pg/ml)
JCM 20101	*Lactococcus lactis* subsp.*lactis*	212.53
JCM 5805	*Lactococcus lactis* subsp.*lactis*	187.62
NRIC 1150	*Lactococcus lactis* subsp.*lactis*	113.00
JCM 1180	*Lactococcus lactis* subsp.*hordniae*	95.03
NBRC 100934	*Lactococcus garvieae*	94.09
NBRC 12007	*Lactococcus lactis* subsp.*lactis*	86.87
NBRC 12455	*Leuconostoc lactis*	86.67
NRIC 1540	*Leuconostoc lactis*	75.32
TA-45	*Streptococcus thermophilus*	74.55
JCM 11040	*Lactococcus lactis* subsp.*hordniae*	64.42
NBRC 100676	*Lactococcus lactis* subsp.*cremoris*	62.41
JCM 5886	*Pediococcus damnosus*	58.31
JCM 16167	*Lactococcus lactis* subsp.*cremoris*	50.35

### Effect of L. Lactis JCM5805 and JCM20101 on mDC and pDC Activation

**Figure 1:**
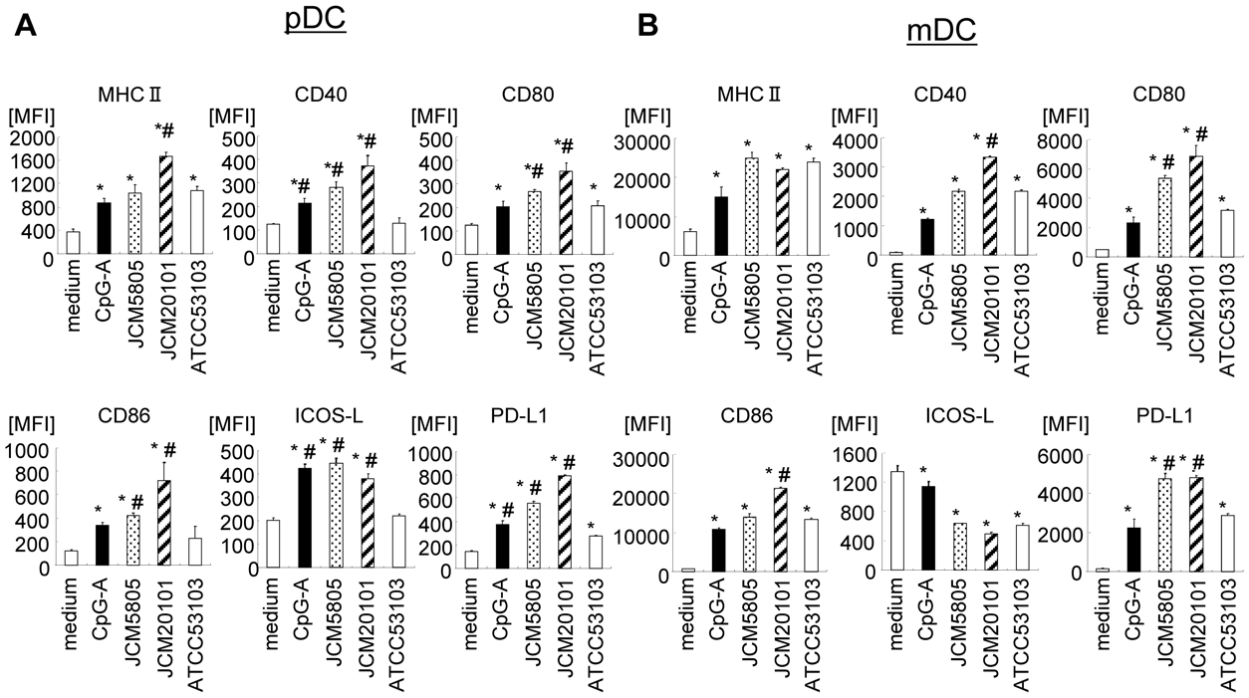
Comparison of DC activation by *L. lactis* and *L. rhamnosus*. Flt-3L induced BM-derived DC were treated with 10 µg/ml of stimulatory (*L. lactis* JCM5805 or JCM20101) or non-stimulatory (*L. rhamnosus* ATCC53103) LAB strains; 0.1 µM of CpG-A was included as a positive control. After 48 hrs, cells were evaluated for expression level of cell-surface markers by flow cytometry. A. Gated on pDC. B. Gated on mDC. Numbers in graph indicates Median Fluorescent Intensity (MFI). All test conditions were significantly elevated (p<0.05) compared to medium control, except for ICOS-L for mDC (B). *, p<0.05 for comparison to medium control; #, p<0.05 for comparison to *L. rhamnosus* ATCC53103. Representative data from three independent experiments are shown. Each experiment was done with triplicate cultures; data are mean ± SD for triplicate cultures.

In order to detail the effects of selected LAB strains on DC activation, we evaluated BM-derived Flt-3L induced DC cultures, gated on pDC and mDC subsets, for changes in cell surface markers,and production of IFNs. DC cells were cultured with two representative spherical strains, *L. lactis* JCM5805 and JCM20101, a representative rod-shaped strain *Lactobacillus rhamnosus* ATCC53103, or the positive control CpG-ODN (Figure 1). We first compared cell surface marker expression on DC, gated at the time of analysis for pDC or mDC. In the pDC population, significant increases (compared to medium control) were induced by the *L. lactis and L. rhamnosus* strains for MHC class II and CD80 (Figure 1A). Although the *L. lactis* strains also increased CD40 and CD86, *L. rhamnosus* had no significant effect on CD40 and CD86 compared to medium control, and there were significant difference between *L. lactis* and *L. rhamnosus* with impact on the co-stimulatory molecules CD40, CD80 and CD86. Further differenced between *L. lactis* and *L. rhamnosus* were observed for cell surface molecules involved in immune suppression. ICOS-L and PD-L1, which are reported to induce Treg [40], [41]. These were greatly up-regulated by *L. lactis*. In contrast, *L. rhamnosus* had only a minor effect on PD-L1 and no effect on ICOS-L. Similar results were observed for mDC, with the interesting exception that ICOS-L was constitutively elevated in mDC; this expression was reduced by treatment with both *L. lactis* and *L rhamnosus* (Figure 1B).

**Figure 2:**
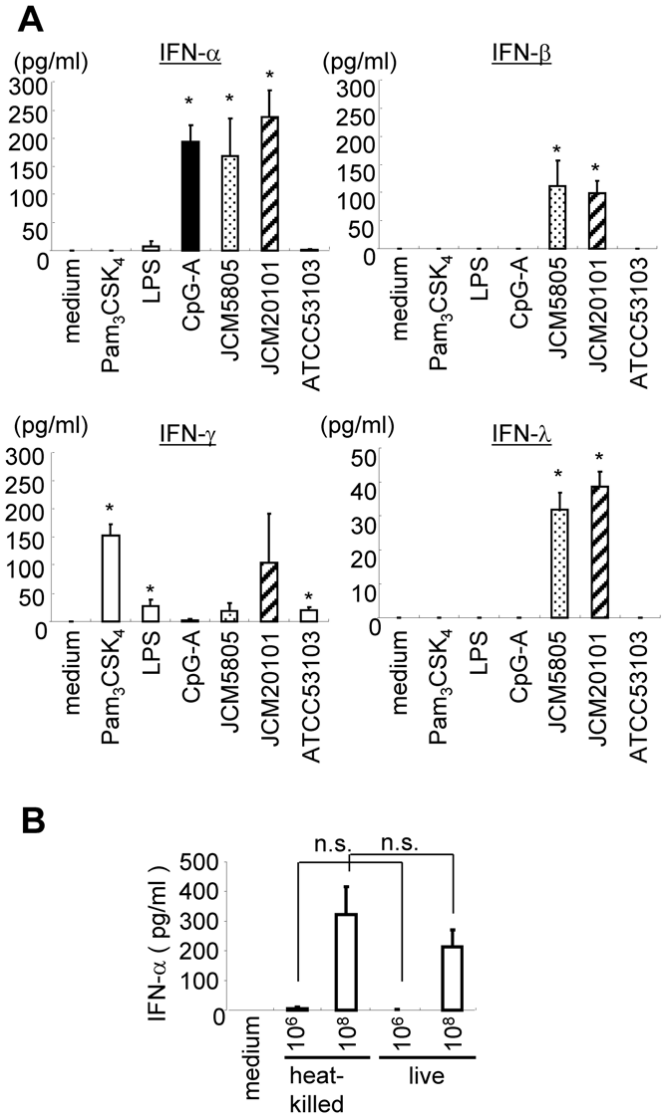
Production of IFNs by Flt-3L induced DC in response to *L. lactis* and *L. rhamnosus*. A. pDC and mDC mixed Flt-3L BM DC were stimulated with 10 µg/ml of *L. lactis* JCM5805, JCM20101 and *L. rhamnosus *ATCC53103 for 48 hrs. 1 µg/ml of Pam_3_CSK_4_ (TLR2L), 5 ng/ml of LPS (TLR4L), 0.1 µM of CpG-A (TLR9L) were used as positive controls. Culture supernatants were assayed for IFN-α, IFN-β, IFN-γ and IFN-λ by ELISA. B. pDC and mDC mixed Flt-3L BM DC were stimulated with heat-killed or live JCM5805 strain. 10^6^ or 10^8^ cells of JCM5805 were added to culture, respectively. Culture supernatants were assayed for IFN-α. Representative data from three independent experiments are shown. Each experiment was done with triplicate cultures; data are mean ± SD for triplicate cultures. *, p<0.05 for comparison to medium control. n.s  =  no significant difference.

We next evaluated the effect of *L. lactis* and *L rhamnosus* treatment on DC production of IFNs (IFN-β, IFN-γ and IFN-λ). As shown in Figure 2A, both IFN-β and IFN-λ1 were induced by two *L. lactis* strains, but this was not observed with *L. rhamnosus*. Unlike IFN-α, positive control CpG ODN could induce neither IFN-β nor IFN-λ1 at the concentrations tested. The type 2 interferon IFN-γ was significantly induced by *L. rhamnosus*, but this was not observed with *L. lactis* strains. The results with IFN-α, IFN-β and IFN-λ were selectively up-regulated by *L. lactis* strains. This is consistent with the previous observation that type I and type III IFNs are typically coordinately regulated [42]. It thus appears that these *L.lactis* strains are potent inducers of DC activation with respect to type I and III IFNs. To confirm whether activity of LAB strains differed between live and heat-killed cells, the same numbers of live or heat-killed *L.lactis* JCM5805 were tested for stimulation of IFN-α production. As shown in Figure 2B, there was no statistical difference in IFN-α stimulation by live and heat-killed JCM5805.

### Cooperation between pDC and mDC Involved in IFN-α Production Induced by *L. Lactis*

**Figure 3:**
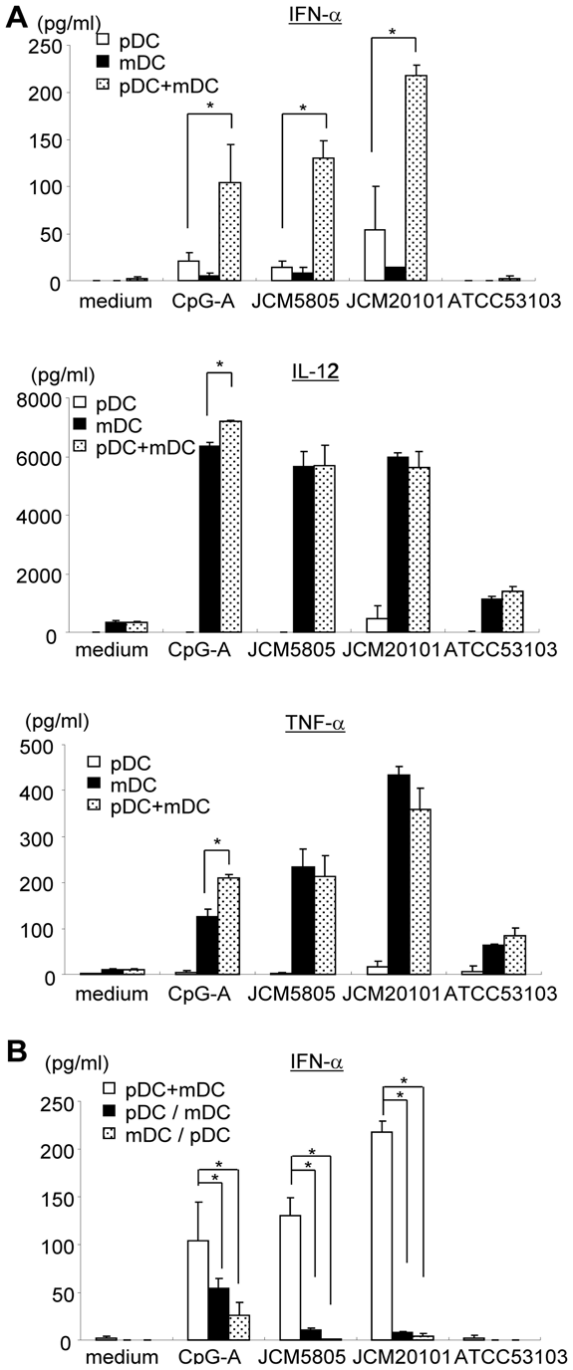
LAB-induced cytokines production in purified pDC, mDC and mixed cultures. A. pDC and mDC were sorted from Flt-3L induced BM-derived DC. 10 µg/ml of *L. lactis* JCM5805, JCM20101 and *L. rhamnosus* ATCC53103 were added to pDC, mDC or pDC and mDC mixed at same ratio (pDC+mDC) for 48 hrs. Concentrations of IFN-α, IL-12 and TNF-α were determined by ELISA. *, p<0.05 for comparison to pDC alone (IFN-α) or to mDC alone (IL-12 and TNF-α). B. Effect of inhibition of direct pDC-mDC contact by transwell system. “pDC+mDC” indicates same number (1×10^5^ cells each) of pDC and mDC cultured together. “pDC/mDC“ indicates pDC and LABs added to the upper chamber, and mDC added to the lower chamber. “mDC/pDC” indicates mDC and LABs added to the upper chamber, and pDC added to the lower chamber. *, p<0.05 for comparison to pDC+mDC. Representative data from three independent experiments are shown. Each experiment was done with triplicate cultures; data are mean ± SD for triplicate cultures.

Since Flt-3L induced DC represent a mixed culture of pDC and mDC, we wondered whether *L. lactis* JCM5805 and JCM20101 stimulated pDC directly or in association with mDC. To address this point, IFN-α production was examined using purified pDC, purified mDC, or mixed pDC/mDC cultures (Figure 3A). *L. lactis* strains induced weak IFN-α production from purified pDC, and no production was observed with *L. rhamnosus*. Interestingly, the amount of IFN-α was dramatically increased when pDC was cultured in the presence of same number of mDC. These data imply that *L. lactis* strains stimulated pDC directly, although maximum effects occurred when mDC and pDC mutually interacted. In contrast, production of IL-12p40 and TNF-α, common cytokines produced by mDC, were completely dependent on the presence of mDC, and synergistic effects with pDC were not detected.

To explore the mechanism of augmented production of IFN-α elicited by *L. lactis* when pDC and mDC were co-cultured, pDC and mDC were separately cultured using a transwell system. When pDC and mDC were separated in the upper and lower chambers, and LAB strains were added to the upper chamber (pDC/mDC or mDC/pDC), the synergistic effect on IFN-α production was completely abrogated (Figure 3B). Therefore, cell-cell contact between pDC and mDC was required for the synergistic effect on *L. lactis-*induced IFN-α production. In contrast, while CpG-ODN also induced the highest IFN-α production when pDC and mDC were cultured together, significant production occurred when pDC and mDC were separated. This data suggests that LAB and CpG-ODN induced IFN-α production were distinguishable in cellular interaction requirements.

### Microscopic Observation of pDC Stimulated by *L. Lactis*

**Figure 4:**
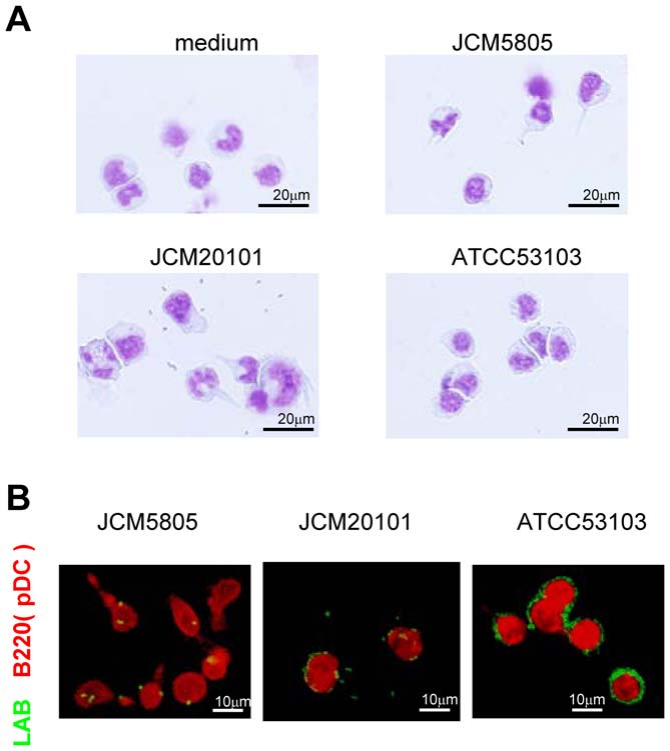
Morphology of pDC stimulated by LABs and incorporation into pDC. A. *L. lactis* JCM5805, JCM20101 and *L. rhamnosus* ATCC53103 were added to pDC for 48 hrs, respectively. Cells were placed onto slide grass by cytospin and stained by Diff-Quick. B. FITC-stained *L. lactis* JCM5805, JCM20101 and *L. rhamnosus* ATCC53103 were added to BM-derived pDC, and were subsequently incubated for 24 hrs. Cells were stained with PE-Cy5.5 labeled anti-B220 antibody as pDC marker, and imaged by fluorescence microscopy.

*L. lactis* strains JCM5805, JCM20101 and *L. rhamnosus* strain were added to pDC cultures and characterized microscopically. As shown in Figure 4A, dendrite formation in pDC was elicited by the *L. lactis* strains, but not by *L. rhamnosus*. To test whether there might be differences between *L. lactis* and *L. rhamnosus* in terms of recognition by pDC, fluorescent dye-labeled LAB strains were added to pDC, and the efficiency of LAB uptake was assessed. As shown in Figure 4B, *L* *. lactis* strains were incorporated into pDC within 24 hours, whereas *L. rhamnosus* was not. *L. lactis* strains accumulated cytoplasmically, while *L. rhamnosus* strain remained attached to the pDC cell surface without internalization. These data suggest that the difference in cytokine induction and morphologic maturation by *L. lactis* versus *L. rhamnosus* might reflect their distinct recognition and compartmentalization by pDC.

**Figure 5:**
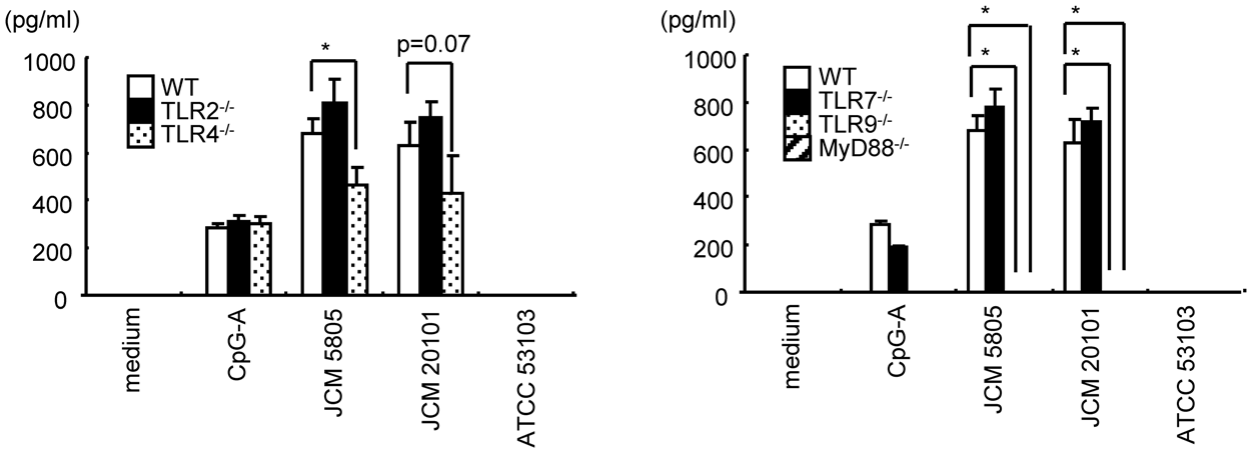
Role of TLR signaling in IFN-α production elicited by LAB. Flt-3L induced BM-derived DC were prepared from wild-type (WT), TLR2^-/-^ and TLR4^-/-^ (left panel), or WT, TLR7^-/-^, TLR9^-/-^ and MyD88^-/-^ (right panel). Cells were cultured in the presence of 0.1 µM of CpG-A or 10 µg/ml of LAB for subsequent 48 hrs. Representative data from three independent experiments are shown. Each experiment was done with triplicate cultures; data are mean ± SD for triplicate cultures. *, p<0.05 for comparison to wild-type.

**Figure 6:**
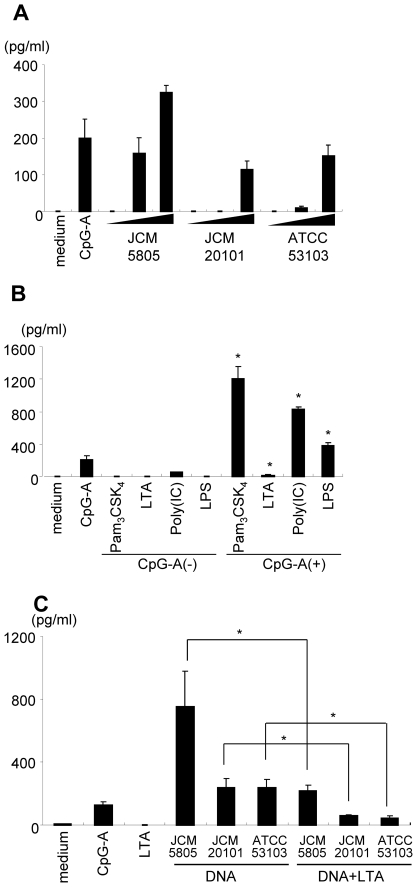
Effect of LAB-derived nucleic acids and combination with TLRLs on IFN-α production. A. DNA was extracted from *L. lactis* JCM5805, JCM20101 and *L. rhamnosus* ATCC53103. Each sample was added at the concentrations of 1, 5, and 10 µg/ml, respectively, to Flt-3L induced BM-derived DC. B. TLR2Ls (1 µg/ml Pam_3_CSK_4_, 10 µg/ml LTA), TLR3L (10 µg/ml Poly(I:C)) and TLR4L (5 ng/ml LPS) in combination with 0.1 µM of CpG-A and IFN-α production were measured. *, p<0.05 for comparison to CpG-A alone. C. Effect of LTA on IFN-α production induced by LAB-derived DNA. 10 µg/ml of LTA was mixed with 10 µg/ml of DNA prepared from *L. lactis* JCM5805, JCM20101 or *L. rhamnosus* ATCC53103 and added to Flt-3L induced BM-derived DC. *, p<0.05 for comparison to LAB-derived DNA alone. Representative data from three independent experiments are shown. Each experiment was done with triplicate cultures; data are mean ± SD for triplicate cultures.

### TLR/MyD88 Signaling Responsible for IFN-α Production Induced by *L. Lactis*

In order to determine the responsible recognition system leading to IFN-α induction by *L. lactis*, Flt-3L DC cultures were evaluated in selected TLR or MyD88 knockout mouse strains. As shown in Figure 5, IFN-α production induced by *L. lactis* strains was completely abrogated both in TLR9^-/-^ and MyD88^-/-^ mice. TLR9 has long been considered as a bacterial sensor based on its specificity for unmethylated CpG-rich DNA, a characterstic molecular feature of prokaryotes. Accordingly, DNA was prepared from LAB strains, and tested for stimulation of IFN-α production in wild-type Flt-3L DC culture. As shown in Figure 6A, DNA prepared from *L. lactis* JCM5805, JCM20101 and *L. rhamnosus* ATCC53103 induced significant IFN-α production at 10 µg/ml. As noted above, *L. rhamnosus* ATCC53103 did not induce IFN-α production when added as whole bacteria. However, isolated DNA from this strain efficiently induced IFN-α, suggesting that *L. rhamnosus* ATCC53103 is not recognized by pDC encountered as whole bacteria. Interestingly, IFN-α production induced by *L. lactis* strains was partially decreased in TLR4^-/-^, suggesting a contribution of TLR4L, presumably through recognition of a cell wall component (Figure 5).

Since complete impairment in TLR9^-/-^ and partial decrease in TLR4^-/-^ were observed, cooperation between TLR4 and TLR9 signaling in IFN-α production was suspected. Therefore, we tested for a synergistic effect of TLR4L and TLR9L. TLR2Ls, which are abundant in the Gram-positive bacterial cell wall, were also examined in combination with the TLR9L CpG ODN. As shown in Figure 6B, Pam_3_CSK_4_, Poly(I:C) and LPS alone could not induce IFN-α, but a striking elevation of IFN-α was observed when they were added in combination with CpG ODN compared to CpG ODN alone. This data suggests that TLR2L, TLR3L or TLR4L synergistically work with CpG ODN in IFN-α production. In contrast, the TLR2L, lipoteichoic acid (LTA) decreased IFN-α production when it was added with CpG-ODN. To further test this effect, LTA was mixed with LAB-derived DNA, and IFN-α production was compared with LAB-derived DNA alone. In accord with CpG ODN, IFN-α production elicited by *L. lactis* or *L. rhamnosus* DNA also was strongly inhibited by lipoteichoic acid (Figure 6C). This data indicate that different TLR2L compounds can either promote or inhibit the effect of TLR9L on IFN-α production. While the basis of this discordance is uncertain, it may be attributable to either contaminating TLRLs sometimes present in TLR2 preparations; or, distinct co-receptors required by these two TLR2Ls (lipoteichoic acid, but not Pam_3_CSK_4_) requires LBP and CD14 interaction [43].

### Treg Induction by pDC Treated by *L. Lactis*

**Figure 7:**
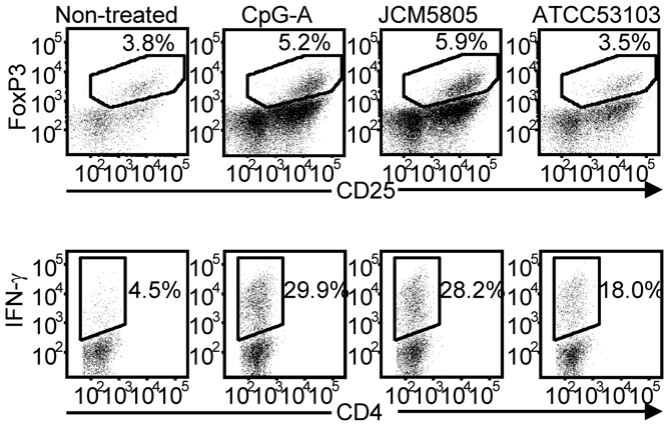
CD4^+^CD25^+^FoxP3^+^Treg and IFN-γ producing CD4^+^T cell generation by *L. lactis* JCM5805-treated pDC. MLR reactions using naïve CD4^+^T cell prepared from BALB/c and purified C57BL/6 derived-pDC stimulated by *L. lactis* JCM5805 (10 µg/ml) or *L. rhamnosus* ATCC53103 (10 µg/ml) or CpG-A (0.1 µM) was performed. After 7 days, cells were collected and stained for induced Treg cells (Data was gated for CD3^+^CD4^+^ cells, and graphed for CD25 and FoxP3 expression) or IFN-γ producing CD4^+?^T cells (Data was gated for CD3^+^ cells, and graphed for CD4 and IFN-γ expression). A representative dataset from three independent experiments with singlet culture is shown.

ICOS-L and PD-L1 (Figure 1A), which were strongly induced by *L. lactis* strains, are reported to be involved in Treg development. Therefore, we tested the effect of *L. lactis* on the induction rate of CD4^+^CD25^+^FoxP3^+^ Treg by pDC in the setting of allogeneic mixed lymphocyte cultures. BM-derived C57BL/6 pDC were purified and stimulated by *L. lactis* JCM5805, *L. rhamnosus* ATCC53103, or a CpG-ODN positive control. Those stimulated pDC were then co-cultured with allogenic naïve BALB/c CD4^+^ T cells, and levels of Treg formation were assessed (Figure 7). Naïve CD4^+^T cell co-cultured with non-treated pDC failed to form Treg cells. However, pDC cultured with LAB strains or CpG were proficient for inducing Treg formation. Moreover, *L. lactis* stimulated pDC yielded 1.7-fold higher CD4^+^CD25^+^FoxP3^+^ Treg compared with *L. rhamnosus* stimulated pDC and similar tendency was observed by CpG-treated pDC. These findings are consistent with the prediction that the selective induction of ICOS-L or PD-L1 by *L. lactis* (versus *L. rhamnosus)* may contribute enhancement of Treg induction by pDC. We also evaluated the ratio of effector T cells induced by stimulated pDC. IFN-γ producing CD4^+^T cells induced by *L. lactis* stimulated pDC were similar to CpG-treated pDC, and 1.6-fold increased compared to *L.rhamnosus* stimulated pDC.

### Effect of Oral Administration of *L. Lactis* JCM5805 on pDC *in Situ*

**Figure 8:**
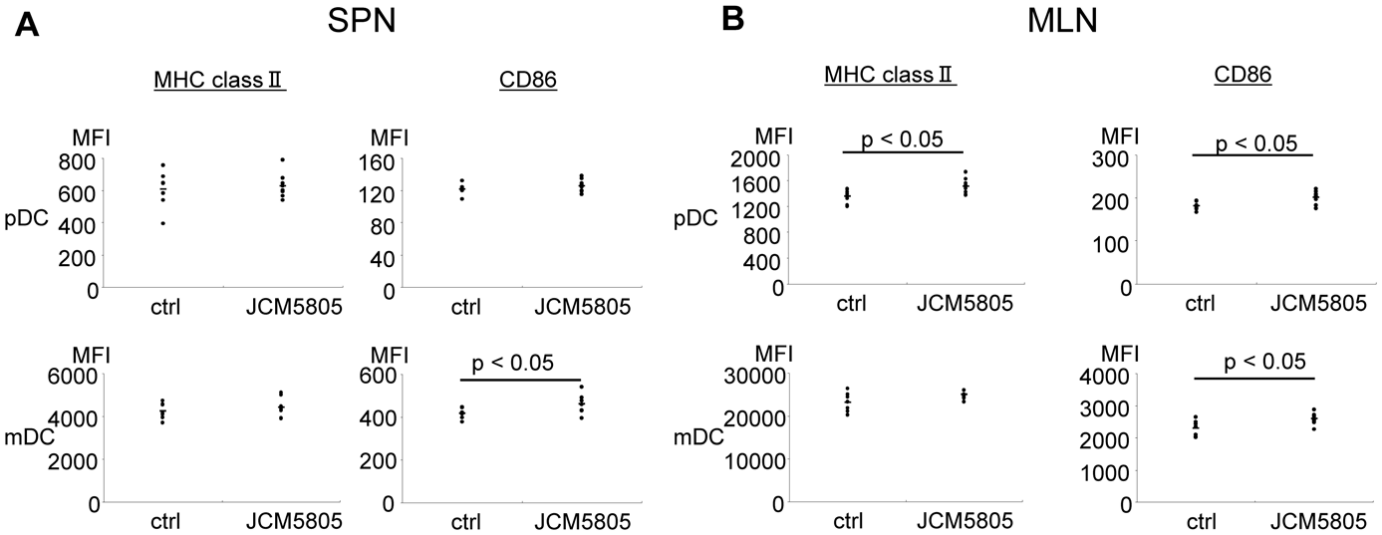
Effect of oral administration of *L. lactis* JCM5805 to healthy C57BL/6 mice. Healthy C57BL/6 mice were divided into two groups (n = 8 each). Control group (ctrl) were fed a normal diet, and *L. lactis* group (JCM5805) were fed a diet containing 1 mg of JCM5805 per day. Two weeks later, low density cells fractions prepared from SPN (A) or MLN (B) derived each group were analyzed for MHCII and CD86 on pDC or mDC. Representative data from three independent experiments are shown.

Since pDC are particularly rare in the intestine, and immature pDC lack dendrites to incorporate materials from mucosal surface, it is uncertain if pDC *in vivo* indeed encounter luminal LAB resulting from dietary intact. However, enteric bacteria and their products undergo uptake and transfer to draining mesenteric lymph nodes (MLN) by several mechanisms. So, we hypothesized that the response to dietary LAB might be detectable in MLN-resident pDC. To assess this idea, wild-type C57BL/6 were fed 1 mg/mouse/day of *L. lactis* JCM5805, and the activation status (elevated MHC class II and CD86) of DCs were determined after two-week administration. DCs were evaluated in the spleen (representative of a negative control, remote systemic lymphoid site), and the MLN (representing the lymphoid site directly draining the intestinal tract, where LAB and their products would accumulate from oral administration). As shown in Figure 8, oral administration of *L. lactis* JCM5805 did not effect MHCII and CD86 expression in pDC located in the spleen. In contrast, a small but statistically significant increase of MHCII and CD86 were observed on pDC located in the MLN. Regarding mDC, JCM5805 induced an increase in CD86 both spleen and MLN derived from JCM5805 group; curiously, this was not accompanied by any change in MHCII expression. These data suggest that small amounts of *L. lactis* JCM5805 administrated orally might be taken up from the intestinal tract, and indeed activate pDC at draining mucosal sites.

## Discussion

The present study is the first demonstration that certain LAB strains commonly used in food products directly stimulate pDC to produce type I and type III IFN. It was intriguing that only select spherical LAB strains stimulated IFN-α production. This was analyzed in detail with strains of *L. lactis* versus the bacillary species, *L. rhamnosus*, raising the simple possibility that pDC may sense size or shape of bacteria. Most studies of bacterial IFN induction in DC involve pathogenic intracellular and extracellular bacteria, and such induction was not observed in the pDC subset, perhaps due to their deficiency in bacterial uptake [44]. Among bacterial pathogens, the spherical bacterium *S. aureus* was shown to activate pDC directly [27]. In the case of *Helicobacter pylori, a* change in its morphology from spiral-shaped into coccoid formis was observed upon phagocytosis by resident dendritic cells located in Peyer’s patch [45]. However, since only select spherical bacterial species induce pDC activation, other factors besides morphology are obviously involved. In a series of knockout mouse experiments, we demonstrated that *L. lactis* stimulation of pDC IFN-α production involved a key role for TLR9 in the context of MyD88 signaling, and a complementary role for TLR2 or TLR4 sensing. In this respect, the stimulatory LAB strains might be considered an immunomodulatory “drug-delivery system”, with an interior TLR9 ligand and “outer envelope” bearing TLR2 or TLR4 ligands.

The synergism of mDC interaction on pDC IFN-α production was intriguing. Most LAB strains stimulate NK cell, macrophage and mDC function, hence affecting diverse cytokines and cell-surface molecules which affect IFN-α. For example, CD40 and IFN-γ synergistically increase IFN-α production [46]. IFN-γ is a typical cytokine increased by LAB administration, due to its direct, strong induction of IL-12, leading to IFN-γ by Th1 and NK cells [28], [29], [32]. CD40 is a commonly induced receptor in activated mDC and macrophages, and is up-regulated by LAB [32]. Interaction between CD40 on conventional DC and CD40 ligand on pDC can lead to IL-12 production by mDC [47], and such crosstalk may partly explain the synergy of mDC coculture for pDC production of IFN-α.

A number of studies have mentioned the effect of LAB strains on immune activation including IFN-α production. Kitazawa et al. showed that *Lactobacillus* strains induce IFN-α from macrophages [48], and Izumo et al. observed that intranasal or oral administration of *L. pentosus* strain resulted in enhancement of IFN-α production elicited by viral infection, a response in part attributable to the pDC subpopulation [49], [50]. In present study, we have shown that *L. lactis* strains are able to induce not only type I IFN but also IFN-λ. IFN-λ is known to induce identical signal transduction to type I IFN, although its role is critical especially in the mucosal site because its receptor is preferentially expressed on epithelial cells [51]. Therefore, administration of those LAB strains might be effective not only systemic infectious virus but also intestinal viral infection such as rotavirus.

The *in vivo* compartment for encounter of pDC with dietary LAB or their products is uncertain. pDC are quite rare in the intestine itself, where they also lack dendrites, and in their quiescent state, show poor phagocytosis of foreign antigens. We therefore hypothesized that LAB or their products, like enteric commensal microbiota, might undergo intestinal uptake and transfer to the directly draining MLN, where resident pDC would undergo LAB encounter. When it was administrated orally for two weeks to healthy mice using JCM5805 as a representative of pDC-stimulatory strain, activation markers were modestly but significantly elevated in pDC resident in MLNs. In contrast, no such activation was seen in pDC resident in a remote lymphoid site (spleen). Curiously, a distinct mode of mDC activation was observed resident in both draining and remote lymphoid sites. These observations support our hypothesis, although more work must be done to validate the LAB trafficking pathway from enteric lumen, and the lymphoid sites and DC subsets responding to oral LAB *in vivo*.

The pDC population has diverse immunomodulatory roles, ranging from the enhancement of anti-viral immunity (notably through IFN-α production) to augmentation of differentiation of CD4^+^ induced Treg cells [40], [41]. Paradoxically, IFN-α itself interferes with survival of immunoregulatory CD4^+^CD25^+^Treg [52], and *in vivo*, promotes autoimmune and chronic inflammatory diseases [53]. However, the present study revealed that stimulation of pDC with IFN-α inducing *L. lactis* augmented the capacity of pDC to induce CD4^+^CD25^+^Treg generation. This may in part reflect the capacity of these *L. lactis* strains to up-regulate pDC expression of ICOS-L and PD1-L, which are important for Treg generation [41], [42]. Conversely, *L. lactis* treated pDC also induce Th1 differentiation, at levels comparable to CpG-treated pDC [54]. It is thus possible that the immunomodulatory effects of *L. lactis* on pDC may involve overlapping mechanisms with CpG. Thus, it is possible that *L. lactis* stimulation of pDC results in an integrated immunomodulatory response that may both augment anti-viral immunity and attenuate chronic inflammatory states that may be beneficial for promoting tissue repair after infection-mediated injury.

## Supporting Information

Table S1 **List of LAB strains used in this study (rod-shaped strains).**
(DOC)

Table S2 **List of LAB strains used in this study (spherical strains).**
(DOC)
