# Supplementary material for: Spherical Lactic Acid Bacteria Activate Plasmacytoid Dendritic Cells Immunomodulatory Function via TLR9-Dependent Crosstalk with Myeloid Dendritic Cells
Source: PLoS One. 2012 Apr 10;7(4):e32588. doi: 10.1371/journal.pone.0032588 (PMC3323594; doi:10.1371/journal.pone.0032588)
Supplement: Table S1 — List of LAB strains used in this study (rod-shaped strains). (DOC) [file pone.0032588.s001.doc]

Table S1 List of LAB strains used in this study (rod-shaped strains)

| Strain ID | Genera | Culture collection |
| --- | --- | --- |
| JCM 1558 | *Lactobacillus pentosus* | JCM |
| JCM 8333 | *Lactobacillus pentosus* | JCM |
| JCM 8334 | *Lactobacillus pentosus* | JCM |
| JCM 8335 | *Lactobacillus pentosus* | JCM |
| JCM 8336 | *Lactobacillus pentosus* | JCM |
| JCM 8337 | *Lactobacillus pentosus* | JCM |
| JCM 8338 | *Lactobacillus pentosus* | JCM |
| JCM 8339 | *Lactobacillus pentosus* | JCM |
| JCM 8340 | *Lactobacillus pentosus* | JCM |
| ATCC 8014 | *Lactobacillus plantarum* | ATCC |
| ATCC 14917 | *Lactobacillus plantarum* | ATCC |
| JCM 1149 | *Lactobacillus plantarum* | JCM |
| NRIC 1596 | *Lactobacillus plantarum* | NRIC |
| JCM 1112 | *Lactobacillus reuteri* | JCM |
| ATCC 53103 | *Lactobacillus rhamnosus* | ATCC |
| IFO 3425 | *Lactobacillus rhamnosus* | IFO |
| JCM 1136 | *Lactobacillus rhamnosus* | JCM |
| JCM 1157 | *Lactobacillus sakei* | JCM |
| JCM 1150 | *Lactobacillus salivarius* | JCM |

| Strain ID | Genera | Culture collection |
| --- | --- | --- |
| JCM 1192 | *Bifidobacterium breve* | JCM |
| JCM 1217 | *Bifidobacterium longum* | JCM |
| JCM 1021 | *Lactobacillus acidophilus* | JCM |
| JCM 1023 | *Lactobacillus acidophilus* | JCM |
| JCM 1028 | *Lactobacillus acidophilus* | JCM |
| JCM 1032 | *Lactobacillus acidophilus* | JCM |
| JCM 1034 | *Lactobacillus acidophilus* | JCM |
| JCM 1038 | *Lactobacillus acidophilus* | JCM |
| JCM 1132 | *Lactobacillus acidophilus* | JCM |
| JCM 1229 | *Lactobacillus acidophilus* | JCM |
| IFO 3960 | *Lactobacillus brevis* | IFO |
| IFO 12005 | *Lactobacillus brevis* | IFO |
| IFO 12520 | *Lactobacillus brevis* | IFO |
| IFO 13109 | *Lactobacillus brevis* | IFO |
| IFO 13110 | *Lactobacillus brevis* | IFO |
| JCM 1059 | *Lactobacillus brevis* | IFO |
| IFO 12004 | *Lactobacillus casei* | IFO |
| JCM 1134 | *Lactobacillus casei* | JCM |
| JCM 8129 | *Lactobacillus casei* | JCM |
| NRIC 1916 | *Lactobacillus casei* | NRIC |
| NRIC 1917 | *Lactobacillus casei* | NRIC |
| NRIC 1936 | *Lactobacillus casei* | NRIC |
| NRIC 1937 | *Lactobacillus casei* | NRIC |
| NRIC 1941 | *Lactobacillus casei* | NRIC |
| NRIC 1942 | *Lactobacillus casei* | NRIC |
| NRIC 1944 | *Lactobacillus casei* | NRIC |
| NRIC 1945 | *Lactobacillus casei* | NRIC |
| NRIC 1946 | *Lactobacillus casei* | NRIC |
| NRIC 1963 | *Lactobacillus casei* | NRIC |
| NRIC 1981 | *Lactobacillus casei* | NRIC |
| JCM 1002 | *Lactobacillus delbrueckii* subsp.*bulgaricus* | JCM |
| JCM 1012 | *Lactobacillus delbrueckii* subsp.*bulgaricus* | JCM |
| NRIC 1962 | *Lactobacillus fermentum* | NRIC |
| JCM 1017 | *Lactobacillus gasseri* | JCM |
| JCM 1130 | *Lactobacillus gasseri* | JCM |
| JCM 1131 | *Lactobacillus gasseri* | JCM |
| JCM 2124 | *Lactobacillus gasseri* | JCM |
| JCM 5813 | *Lactobacillus gasseri* | JCM |
| JCM 5814 | *Lactobacillus gasseri* | JCM |
| JCM 8789 | *Lactobacillus gasseri* | JCM |
| JCM 8790 | *Lactobacillus gasseri* | JCM |
| JCM 1120 | *Lactobacillus helveticus* | JCM |
| JCM 1155 | *Lactobacillus ilgardii* | JCM |
| JCM 1022 | *Lactobacillus johnsonii* | JCM |
| JCM 2012 | *Lactobacillus johnsonii* | JCM |
| JCM 2122 | *Lactobacillus johnsonii* | JCM |
| JCM 5812 | *Lactobacillus johnsonii* | JCM |
| JCM 8791 | *Lactobacillus johnsonii* | JCM |
| JCM 8792 | *Lactobacillus johnsonii* | JCM |
| JCM 8793 | *Lactobacillus johnsonii* | JCM |
| JCM 5818 | *Lactobacillus kefiri* | JCM |
| NRIC 1968 | *Lactobacillus kimuchii* | NRIC |
| NRIC 1969 | *Lactobacillus kimuchii* | NRIC |
| NRIC 1970 | *Lactobacillus kimuchii* | NRIC |
| ATCC 25302 | *Lactobacillus paracasei* | ATCC |
| ATCC 25303 | *Lactobacillus paracasei* | ATCC |
| IFO 3533 | *Lactobacillus paracasei* | IFO |
| JCM 1053 | *Lactobacillus paracasei* | JCM |
| JCM 1109 | *Lactobacillus paracasei* | JCM |
| JCM 1111 | *Lactobacillus paracasei* | JCM |
| JCM 1133 | *Lactobacillus paracasei* | JCM |
| JCM 1161 | *Lactobacillus paracasei* | JCM |
| JCM 1163 | *Lactobacillus paracasei* | JCM |
| JCM 1172 | *Lactobacillus paracasei* | JCM |
| JCM 1181 | *Lactobacillus paracasei* | JCM |
| JCM 1556 | *Lactobacillus paracasei* | JCM |
| JCM 2123 | *Lactobacillus paracasei* | JCM |
| JCM 2769 | *Lactobacillus paracasei* | JCM |
| JCM 8131 | *Lactobacillus paracasei* | JCM |
| JCM 8132 | *Lactobacillus paracasei* | JCM |
| JCM 8133 | *Lactobacillus paracasei* | JCM |
