# Supplementary material for: Spherical Lactic Acid Bacteria Activate Plasmacytoid Dendritic Cells Immunomodulatory Function via TLR9-Dependent Crosstalk with Myeloid Dendritic Cells
Source: PLoS One. 2012 Apr 10;7(4):e32588. doi: 10.1371/journal.pone.0032588 (PMC3323594; doi:10.1371/journal.pone.0032588)
Supplement: Table S2 — List of LAB strains used in this study (spherical strains). (DOC) [file pone.0032588.s002.doc]

Table S2 List of LAB strains used in this study (spherical strains)

| Strain ID | Genera | Culture collection |
| --- | --- | --- |
| JCM 8723 | *Enterococcus casseliflavus* | JCM |
| JCM 8730 | *Enterococcus malodoratus* | JCM |
| NBRC 100934 | *Lactococcus garvieae* | NBRC |
| JCM 16167 | *Lactococcus lactis* subsp.*cremoris* | JCM |
| JCM 20076 | *Lactococcus lactis* subsp.*cremoris* | JCM |
| NBRC 100676 | *Lactococcus lactis* subsp.*cremoris* | NBRC |
| JCM 1180 | *Lactococcus lactis* subsp.*hordniae* | JCM |
| JCM 11040 | *Lactococcus lactis* subsp.*hordniae* | JCM |
| JCM 1158 | *Lactococcus lactis* subsp.*lactis* | JCM |
| JCM 5805 | *Lactococcus lactis* subsp.*lactis* | JCM |
| JCM 12650 | *Lactococcus lactis* subsp.*lactis* | JCM |
| JCM 20101 | *Lactococcus lactis* subsp.*lactis* | JCM |
| JCM 20128 | *Lactococcus lactis* subsp.*lactis* | JCM |
| JCM 20312 | *Lactococcus lactis* subsp.*lactis* | JCM |
| JCM 20399 | *Lactococcus lactis* subsp.*lactis* | JCM |
| NBRC 12007 | *Lactococcus lactis* subsp.*lactis* | NBRC |
| NRIC 1147 | *Lactococcus lactis* subsp.*lactis* | NRIC |
| NRIC 1148 | *Lactococcus lactis* subsp.*lactis* | NRIC |
| NRIC 1150 | *Lactococcus lactis* subsp.*lactis* | NRIC |
| NBRC 12455 | *Leuconostoc lactis* | NBRC |
| NRIC 328 | *Leuconostoc lactis* | NRIC |
| NRIC 1540 | *Leuconostoc lactis* | NRIC |
| NRIC 1575 | *Leuconostoc lactis* | NRIC |
| JCM 6124 | *Leuconoctoc mesenteroides* | JCM |
| NBRC 100496 | *Leuconoctoc mesenteroides* | NBRC |
| JCM 5886 | *Pediococcus damnosus* | JCM |
| JCM 2026 | *Pediococcus pentosaceus* | JCM |
| JCM 5885 | *Pediococcus pentosaceus* | JCM |
| JCM 5890 | *Pediococcus pentosaceus* | JCM |
| JCM 20314 | *Pediococcus pentosaceus* | JCM |
| JCM 20459 | *Pediococcus pentosaceus* | JCM |
| JCM 20026 | *Streptococcus thermophilus* | JCM |
| ST-21 | *Streptococcus thermophilus* | DANISCO |
| TA-40 | *Streptococcus thermophilus* | DANISCO |
| TA-45 | *Streptococcus thermophilus* | DANISCO |
